# Birth-related soft tissue injury due to transverse malpresentation at delivery: a case report

**DOI:** 10.1515/crpm-2023-0009

**Published:** 2023-08-14

**Authors:** Celine Rohaert, Anne Poleij, Chantal Quispel, Miranda de Jong, Pierluigi Ciet, Florian Cassel

**Affiliations:** Division of Neonatalogy, Department of Neonatal and Pediatric Intensive Care, Erasmus MC–Sophia Children’s Hospital, Rotterdam, The Netherlands; Department of Obstetrics and Gynecology, Albert Schweitzer Hospital, Dordrecht, The Netherlands; Department of Obstetrics and Gynecology, Erasmus MC Rotterdam and Albert Schweitzer Hospital, Dordrecht, The Netherlands; Department of Pediatrics, Albert Schweitzer Hospital, Dordrecht, The Netherlands; Department of Radiology and Nuclear Medicine Department and Pediatric Respiratory Medicine, Erasmus MC–Sophia Children’s Hospital, Rotterdam, The Netherlands; Department of Radiology, University of Cagliari, Cagliari, Italy

**Keywords:** birth trauma, fetal malpresentation, mechanical birth-related injury, soft tissue mass, transverse position

## Abstract

**Objectives:**

Birth-related mechanical trauma to the newborn is an important issue and may be underestimated [Chaturvedi A, Chaturvedi A, Stanescu AL, Blickman JG, Meyers SP. Mechanical birth-related trauma to the neonate: an imaging perspective. Insights Imag 2018;9:103–18]. Risk factors for birth-related injuries include vacuum or forceps delivery, large size for gestational age and abnormal presentation before delivery [Gupta R, Cabacungan ET. Neonatal birth trauma: analysis of yearly trends, risk factors, and outcomes. J Pediatr 2021;238:174–80]. When a newborn has a soft tissue mass, there is a wide range of potential diagnoses, ranging from benign traumatic origins to aggressive phenotypes of malignant tumors [Thacker M. Benign soft tissue tumors in children. Orthop Clin N Am 2014;44:433–44]. Diagnosing a congenital tumor in a newborn creates uncertainty for parents and health care providers. Accurate imaging is crucial for distinguishing soft tissue mass origins.

**Case presentation:**

A 32 weeks 6 days pregnant Caucasian woman was admitted after premature prelabor rupture of membranes (PPROM). Fetal ultrasound showed no abnormalities, the infant was born by a caesarean section. The delivery was complicated by the infant’s transverse position. A female infant was born with a large left-sided dorsal soft tissue mass at the thoracic level with elastic consistency, and multiple skin lacerations. A broad differential diagnosis was made. Additional imaging was suggestive for a posttraumatic swelling due to transverse position during birth. The mass decreased and disappeared over three days.

**Conclusions:**

The diagnosis of a soft tissue mass in a newborn can be challenging. A birth-related trauma affecting the soft tissue should be considered, especially if prenatal ultrasound findings were normal. Malpresentation during birth is a significant risk factor. Accurate diagnostic imaging is important to do before conducting further diagnostic examinations. The time course of the mass, before and after birth, can aid in determining its origin.

## Introduction

For large soft tissue masses in newborns, a wide range of diagnoses should be considered. Birth-related soft tissue injury is not always considered first in the differential diagnosis. Discrimination between a benign or a malignant mass is an important first step. The most common benign soft tissue lesions in children are vascular tumors, fibrous and fibrohistiocytic tumors, and pseudotumors [1, 2]. For neonates and infants, the most frequently observed tumors include vascular tumors, fibromatosis colli, infantile myofibromatosis, infantile fibrosarcoma, and subcutaneous metastases from neuroblastoma [2]. Hence these diagnoses are usually the first to be considered. A multidisciplinary approach in a tertiary care center is recommended. Accurate imaging is crucial to swiftly determine the origin of the mass, and to make further diagnostic decisions. In most cases, after initial screening with ultrasound (US), magnetic resonance imaging (MRI) is the most sensitive technique to assess the extent and the possible nature of the lesion [1], [2], [3].

Birth-related injuries can be divided into two categories: mechanical events and hypoxic-ischemic events. The incidence of neonatal mechanical birth-related trauma is 0.82 %, but this is likely underreported [4]. Common forms of traumatic birth injuries are soft-tissue injuries. Vacuum or forceps delivery, large for gestational age, and malpresentation at delivery are well-known risk factors [5]. This case describes an unusual and challenging presentation of a mechanically-related birth injury due to fetal malpresentation. In this paper, the authors illustrate an unusual presentation of a mechanical traumatic birth injury with a challenging and misleading diagnosis.

## Case presentation

A 28-year-old Caucasian woman was admitted to the obstetric department with PPROM at a gestational age (GA) of 32 weeks and six days. At admission the amniotic fluid was clear, there were no uterine contractions and the cardiotocography (CTG) was normal. The ultrasound (US) revealed a diagnosis of anhydramnios, with the fetus in a transverse position with the back in a caudal position, and a cervical length of 43 mm. Screening for Group B Streptococcus was negative. The white blood cell count was 12.5 × 10^9^ mmol/L and the C-reactive protein (CRP) level was found to be 36 mg/L.

Prior to the admission, the pregnancy had been uneventful. Ultrasounds at 12 and 20 weeks GA and the first trimester non-invasive prenatal test (NIPT) showed no abnormalities. Fetal biometry at 30 weeks GA showed normal fetal growth, with fetal abdominal circumference (FAC) at the 69th and estimated fetal weight (EFW) at the 48th percentile.

When admitted to the ward, betamethasone and nifedipine were prescribed for lung maturation and tocolysis was administered during the first two days of admission according the local protocol. At 33 weeks GA, fetal biometry was repeated, which showed reduced growth velocity; with a stable FAC, yet an EFW at the 23th percentile. The pulsatility index of the umbilical artery was normal. While performing the biometry, there were no signs of fetal abnormalities.

On day six of admission, the patient started to have increasing irregular contractions. The fetus became tachycardic without the presence of maternal fever. Tachycardia persisted the next day, with an increase in white blood cell count to 14.6 × 10^9^ mmol/L and CRP 70 mg/L. With the suspicion of chorioamnionitis the next day, a caesarean section was performed at GA 33 weeks and 6 days.

The C-section was complicated by the anhydramnios, the fetus being in transverse position and an unknown large mass on fetus’ back, which was the presenting part in the uterotomy. Assessing the fetus’ exact position was challenging due to the mass. An internal version to either a cephalic or a breech presentation was not possible without expanding the laparotomy and uterotomy. After the extension, the fetus was delivered in a cephalic position.

A female infant, weighing 2945 g (>P97) was born with a moderate start with APGAR scores of one and seven at one and five minutes of life, respectively. The umbilical blood gas showed a venous pH of 7.06 with a base excess of −8.3 mmol/L. The child received five inflation breaths with consequent ventilation for four minutes before sufficient respiratory drive. Despite non-invasive respiratory support, endotracheal intubation was performed due to respiratory insufficiency and intratracheal surfactant (150 mg/kg body weight) was administered due to infant respiratory distress syndrome.

**Figure 1:**
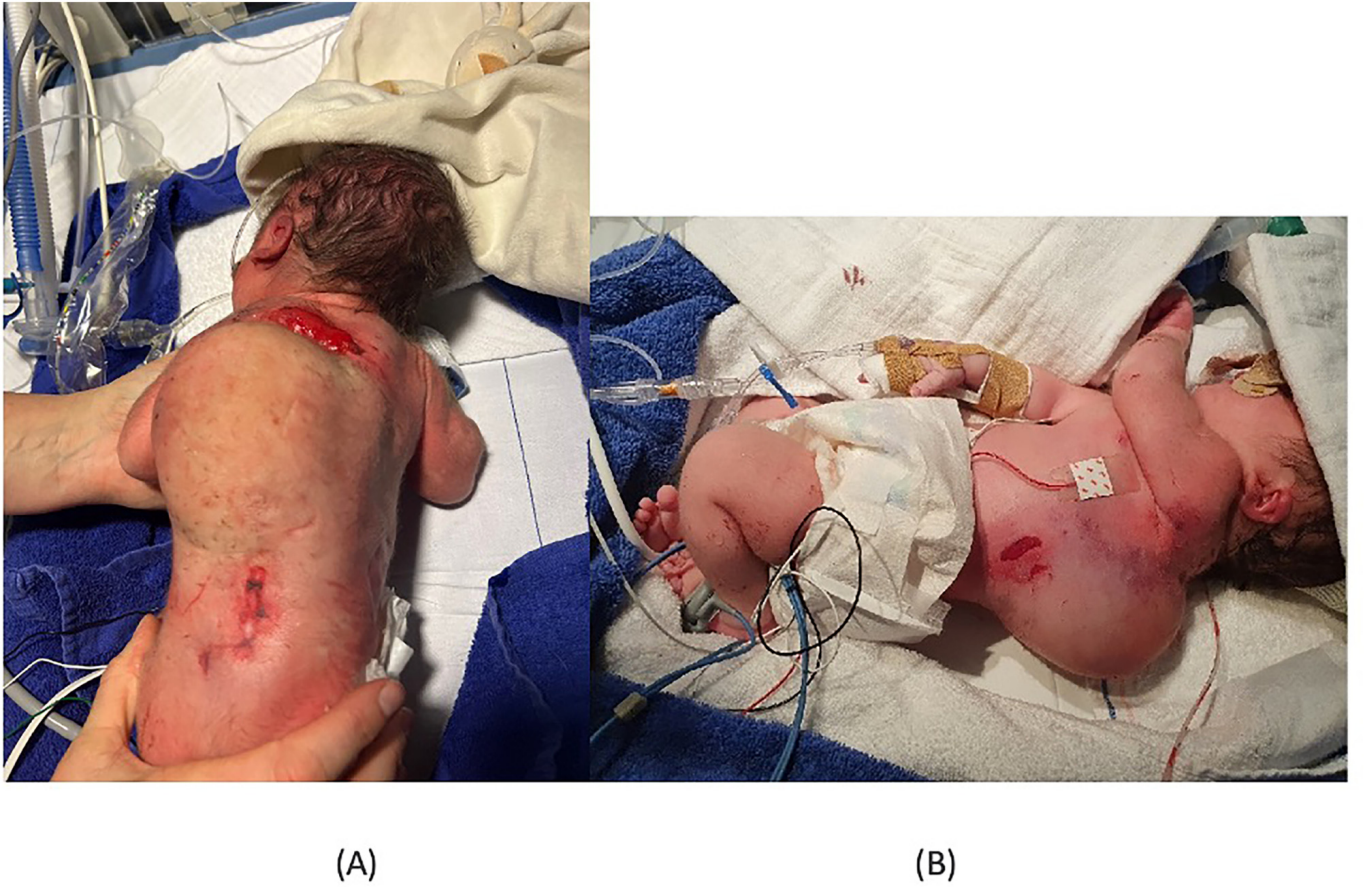
Initial examination. An extensive dorsal mass was remarked at birth at the thoracic level. Open skin lesions were present as well (dorsal (A) and lateral (B) view).

Directly after birth, a prominent dorsal mass of solid-elastic consistence with a diameter of about 12 cm was noticed at the thoracic level (Figure 1). Multiple skin lesions were present around the swelling.

A few hours after birth, a complete blood count and inflammatory parameters showed no abnormalities. Tumor markers were determined and showed a carcinoembryonic antigen (CEA) of 1.59 μg/L (<5 μg/L), cancer antigen (CA) 125 of 12 kU/L (<35 kU/L), beta human chorionic gonadotropin (beta- HCG) of 32.5 IU/L (<1 IU/L), AFP (alpha-fetoprotein) of 1,100,964 ng/mL (5,000–105000 ng/mL). At day 5, beta HCG and AFP were not detectable anymore. Differential diagnosis included a vascular malformation, congenital malignancy, benign tumoral masses like teratoma and hemangioma, and spina bifida occulta.

**Figure 2:**
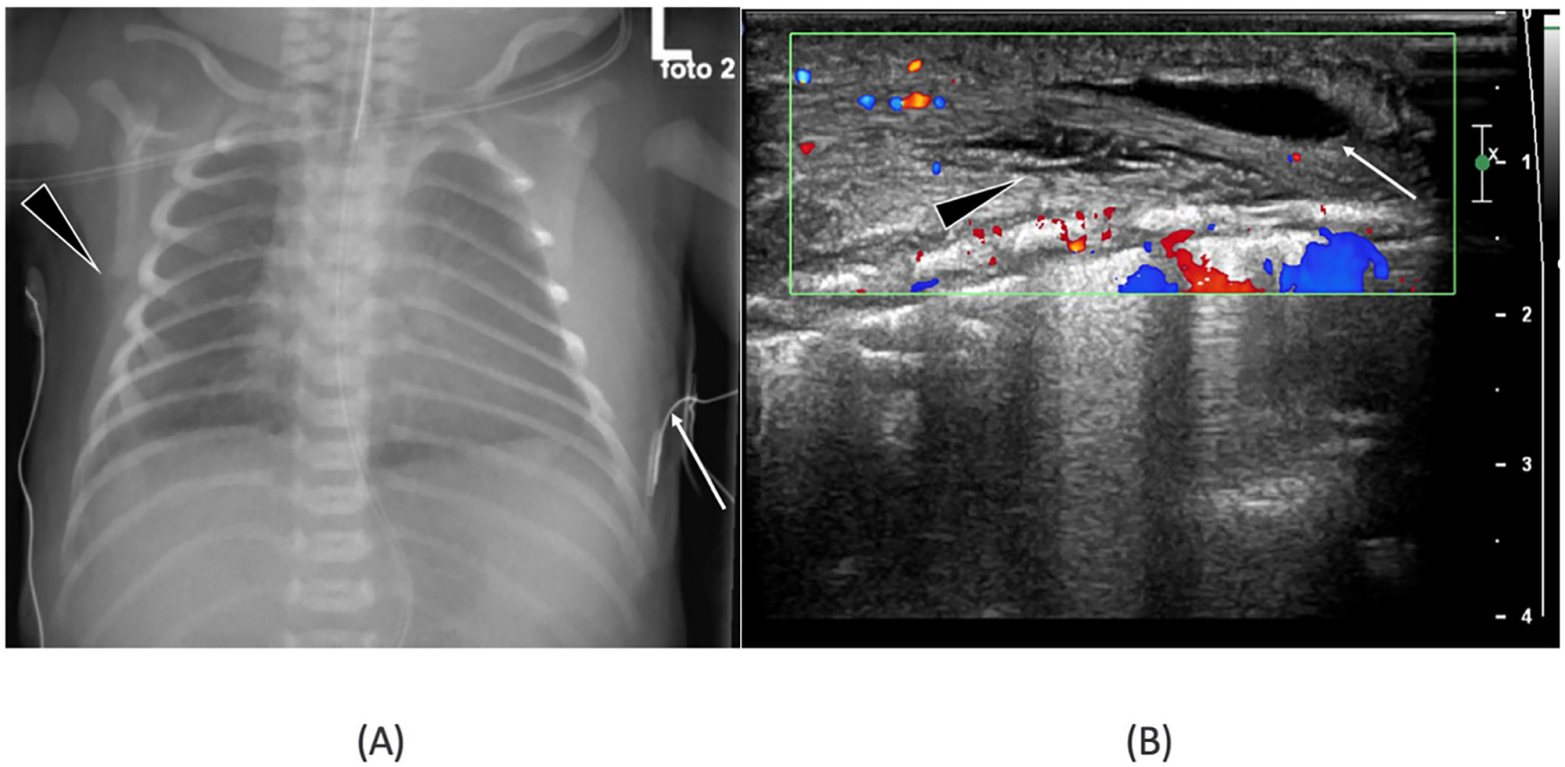
Imaging at day two. Chest wall asymmetry is observed on the left side (arrow) in the AP chest radiograph (CR) compared to the right side (arrowhead) (A). Ultrasound of soft tissue mass with high frequency probe and color doppler technique. US shows fluid collection (arrow) in the subcutaneous fat with separation of planes (arrowhead) (B).

**Figure 3:**
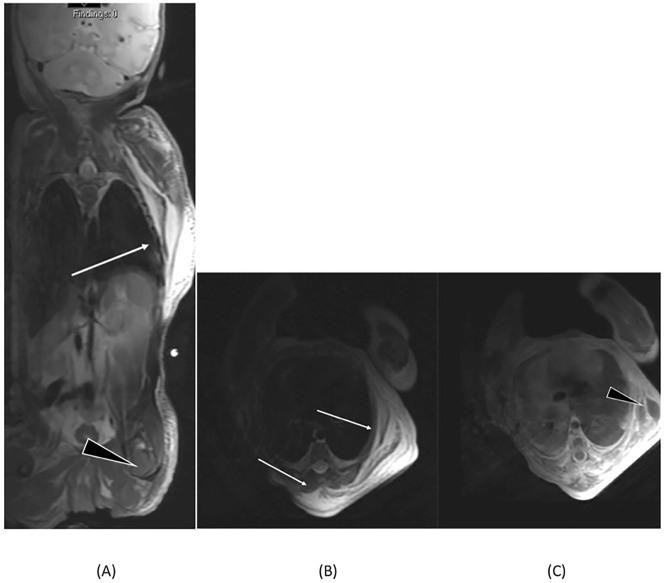
MRI exam. Coronal T2-weighted fat-saturated MRI reveals T2 hyperintense fluid collections (arrow) extending to the left leg’s subcutaneous fat (arrowhead) (A). T2-weighted fat-saturated MRI (B) and T1-weighted post-contrast fat-saturated scan (C) indicate fluid collections (arrow) in the left posterior chest wall without contrast enhancement (arrowhead).

Due to the noticeable mass, further imaging was performed on day two, including chest radiograph (CR), US and MRI. The CR revealed a significant soft tissue asymmetry in the left posterior chest wall (Figure 2A). The US indicated diffuse edema in the subcutaneous fat tissue of the left chest wall and an asymmetrical thickening of the thorax wall muscles (Figure 2B). The MRI revealed that the soft tissue mass was comprised of asymmetrically thickened dorsal thorax wall muscles, particularly the serratus anterior and latissimus dorsi. Additionally, several fluid collections were found in various layers of the subcutis, extending to the left upper limb and to the gluteal muscles (Figure 3A, B). The post-contrast MRI series did not show any enhancing soft tissue masses (Figure 3C). The spontaneous decrease of the mass by the time of imaging was noteworthy and supported the diagnosis of a traumatic origin for the lesion.

**Figure 4:**
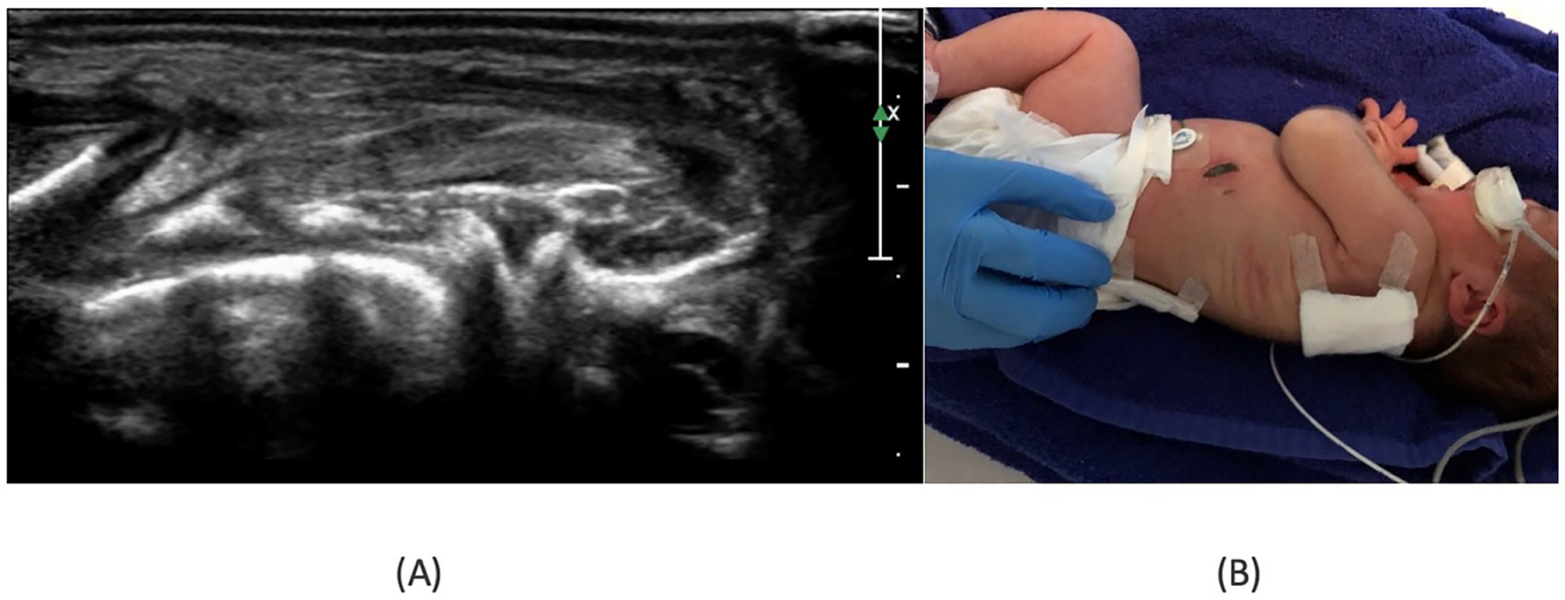
Resolution of soft tissue mass. No fluid collections were noted in the chest wall’s subcutaneous soft tissue at the US six days after birth (A). Disappearance of the clinical swelling at day 8 (B).

On day six, a significant reduction in soft tissue edema, fluid collections, and chest wall musculature thickening was seen, as confirmed by US (Figure 4A). By day eight, the swelling had disappeared clinically (Figure 4B).

The infant was extubated after three days and remained on high flow respiratory support. The weight decreased significantly to 2200 g (P50-90) after a few days, in part due to the decrease in swelling.

Empiric antimicrobial therapy was started at birth because of prematurity and suspected chorioamnionitis and could be stopped after 48 h. At day eight, the child was transferred to a level two hospital for further care because of prematurity.

## Discussion

Birth injury or birth trauma is a structural or functional damage secondary to external forces during labor or delivery [5]. It is an important cause of neonatal morbidity and mortality [4]. The most reported birth injuries are head and skeletal trauma. Most of the time this occurs in the presence of predisposing feto-maternal risk factors. One well-known risk factor is malpresentation at delivery, as in this case. Other risk factors are large for gestational age infants, vacuum/forceps delivery, maternal obesity, and gestational diabetes. The mode of delivery is adapted to these risk factors, with increasing rates of elective cesarean sections, which has been shown to be protective [5].

A Pubmed search was conducted with following MeSH terms: birth injury, labor presentation, fetal malpresentation, soft tissue mass, selected on title and abstract. Little literature is available about transverse malpresentation and especially about possible soft tissue-related injuries. Van der Kaay et al. reported one case, born prematurely in transverse position after PPROM, presenting with a neonatal compartment syndrome of the extremities [6]. No specific cases were found about an extensive dorsal mass.

Fetal malpresentation at birth is defined as any position where the fetal presenting part is other than the vertex. This includes breech presentation, transverse, oblique, face and brow presentation and compound (hand or arm) presentation [7]. Gardberg et al. conducted a retrospective study covering 10 years (1995–2004) with recording of all fetal malpresentations. Nine percent among 11,957 singleton deliveries were malpresentations. Cephalic malposition was the most frequent (5.4 %), 3.1 % had breech presentation and 0.12 % a transverse position. Multiparity increases the likelihood of face presentation and a transverse position [8]. Oblique and transverse position is present in 0.03 % of deliveries. These presentations are most often described in cases where the fetus is small due to prematurity or small for gestational age, or in high parity cases where the uterus is compliant [7]. Preterm birth (<37 weeks GA) accounts for 10 % of all deliveries, with PPROM being present in 30 % of cases. PPROM is also a known risk factor for fetal malpresentation as spontaneous version with PPROM is unlikely. A fetus in transverse position during labor or in mothers with PPROM is at increased risk of lower arterial pH, severe acidosis, and birth trauma such as brachial plexus palsy and extensive ecchymosis [9].

In the case presented here, the fetus was in transverse position and born prematurely by caesarean section after a prolonged period in transverse position, with barely any amniotic fluid and with an episode of irregular contractions. The swelling did not seem traumatic initially because of the large size of the mass.

Both benign and malignant diagnoses were taken into consideration. Soft tissue masses in children are common and include inflammatory and reactive processes, hamartomas and benign and malignant tumors [1]. Taking into account that recent antenatal ultrasound showed no abnormalities, a malignant origin with rapid growth was suspected to be more likely. However, the oligohydramnios could have hindered the ultrasound examination and therefore a mass could have been missed. Malpresentation before birth or trauma were also taken into consideration. The lacerations in our case were suggestive for a traumatic cause, most probably due to the difficult delivery secondary to the dorsal mass. The most evident argument for the benign origin of the mass was the natural decrease over time with complete resolution within eight days. A well-known form of birth related trauma is the caput succedaneum. This is a fluid collection, comparable with the mass in our case, superior to the periosteum, presenting as an edematous, fluctuant, pitting mass caused by pressure from the uterine and vaginal walls on the head during delivery. Bruising, petechiae and ecchymosis may be present as well. Caput succedaneum usually resolves within 48 h, and needs no further ultrasound or imaging. Risk factors for this type of birth trauma include primigravida, PPROM, oligohydramnios, Braxton-hicks contractions, protracted active labor course, and operative vaginal delivery [10].

The determination of the tissue composition of the lesion and localization of the mass are the primary objectives of imaging [2]. MRI is the most important type of imaging for evaluation of soft tissue lesions and gives important details of the anatomy, location and proximity to vital structures [1, 3]. US is more practical and faster, although MRI can be useful especially in the visualization of the deep subfascial soft tissue masses, which are more difficult to evaluate with US [1]. Soler et al. [3] performed a blinded retrospective review of MRI examinations of 65 soft tissue lesions to evaluate the value of each MRI finding in differentiating the nature of soft tissue lesions. Multicompartmental involvement was more common in non-tumoral lesions, while well-defined margins were suggestive for benign lesions. The change from homogeneous to heterogeneous pattern on T1-and T2-weighted sequences was described as predictor of malignancy with a sensitivity of 77 % and specificity of 20 % [3]. Gadolinium contrast can help in differentiating cystic from solid components and showing the degree of vascularity of a lesion [1]. The latter can be useful to differentiate vascular malformations. When a mass of undetermined origin persists, a biopsy is necessary [1, 3]. Imaging with US and MRI in our case were complementary and sufficient to reach the diagnosis. Thickening of the muscle, edema and subcutaneous fluid collections were suggestive of muscle contusion and together could rule out more serious diagnoses. Combining imaging and clinical data can thus aid in diagnosing a congenital soft tissue mass caused by a traumatic event, in this case in the context of prolonged fetal transverse malpresentation in a uterus lacking amniotic fluid and with a history of contractions.

## Take-home messages of lessons learned

Prolonged fetal malposition *in utero*, such as transverse position, can be the origin of a prominent soft tissue injury, although rare. Indirect indications of a traumatic origin are the natural history with spontaneous decrease of the soft tissue mass, the absence of tumoral mass on recent antenatal US, and other superficial lesions of the skin. Accurate imaging using US and MRI is the gold standard, to perform before further diagnostic testing. The prognosis is favorable with complete resolution.
